# Prevalence and clinical characteristics of neurotrophic keratopathy in hispanic population in northeastern Mexico

**DOI:** 10.1007/s10792-023-02726-x

**Published:** 2023-05-04

**Authors:** Alberto Castillo-Macías, Jesús Enrique Arreola-Martínez, Denise Loya-García, Jorge Eugenio Valdez-García

**Affiliations:** Instituto de Ciencias Visuales y Oftalmología, Hospital Zambrano Hellion, TecSalud, Av. Batallón de San Patriciio #112, Real de San Agustín, San Pedro Garza García, Nuevo León México

**Keywords:** Neurotrophic keratitis, Prevalence, Ocular surface, Cornea

## Abstract

**Purpose:**

To evaluate the prevalence and clinical characteristics of neurotrophic keratopathy (NK) in northeastern Mexico.

**Methods:**

Retrospective cross-sectional study in which NK patients admitted to our ophthalmology clinic between 2015 and 2021 were consecutively enrolled. Data regarding demographics, clinical characteristics, and comorbidities were collected at the time diagnosis of NK was made.

**Results:**

In the period from 2015 to 2021, a total of 74,056 patients were treated and of these 42 had a diagnosis of neurotrophic keratitis. The prevalence found was 5.67 [CI95 3.95–7.38] in 10,000 cases. The mean age observed was 59 ± 17.21 years occurring more frequently in males in 59% and with corneal epithelial defects in 66.7%. The most frequent antecedents were the use of topical medications in 90%, the presence of diabetes mellitus 2 in 40.5% and systemic arterial hypertension in 26.2%. A higher proportion of male patients with corneal alterations and a higher proportion of female patients with corneal ulcerations and/or perforation were observed.

**Conclusion:**

Neurotrophic keratitis is an underdiagnosed disease with a broad clinical spectrum. The antecedents that were contracted corroborate what was reported in the literature as risk factors. The prevalence of the disease in this geographical area was not reported, so it is expected to increase over time when searching for it intentionally.

## Introduction

Neurotrophic keratitis (NK) is a degenerative corneal disease characterized by the loss of trigeminal innervation causing hypoesthesia or a total loss of corneal sensitivity. This condition occurs due to ophthalmic nerve fiber damage, which decreases local sensitivity to stimuli and autonomic protective reflexes, resulting in constant epithelial alteration without the ability to regenerate [1].

Given the multiple etiologies and diverse clinical manifestations of neurotrophic keratitis, research on the same has been generally overlooked [2]. This disease has currently been classified as an orphan/rare disease (ORPHA137596), leading to a lack of specific epidemiological data in the literature [3]. Estimates have shown a prevalence and incidence of < 5 per 10,000 inhabitants based on data obtained from diseases and phenomena associated with its development, the most common of which are herpetic keratitis and surgical procedures. Accordingly, herpetic keratitis has an incidence of 6.8 per 100,000 inhabitants per year worldwide, with data from the US showing an incidence of 31.5 per 100,000 population per year and a prevalence of around 150 per 100,000 population per year. One study showed that the prevalence of patients who develop neurotrophic keratitis after herpetic keratitis is 149/100,000 [4]. Moreover, surgical ablation of the trigeminal ganglion has been shown to cause trigeminal nerve injury in 2.8% of patients, leading to a NK prevalence of 0.02/10,000 after surgery [5]. No published data have reported an association between systemic diseases and NK.

One of the characteristics of NK is the decreased production of tears [6, 7], which function to provide a surface that allows for regular light refraction, maintain the metabolism of the ocular surface, and lubricate the ocular surface to facilitate blinking. Tears are composed of water (98.3%), salts (1%), proteins/glycoproteins (0.7%), carbohydrates, lipids, and enzymes [8]. Notably, tear proteins consist of immunoglobulins and neuropeptides that regulate corneal and conjunctival epithelial cell proliferation, migration, and differentiation; exhibit antimicrobial properties; and stimulate lymphocyte and macrophage secretion of enzymes such as peroxidase, lactoferrin, and lysozyme. The complex interactions between the corneal epithelium, tears, and nerve stimulation are essential for proper epithelial function and regeneration. Alterations in corneal sensitivity generate an imbalance in these complex relationships, thereby promoting pathological changes observed in NK [9–11].

### Clinical characteristics

NK usually indicates the presence of congenital or iatrogenic systemic or ocular diseases culminating in damage to the fifth cranial nerve [1]. The most common causes of loss of corneal sensitivity include herpetic keratitis, chemical burns, prolonged use of contact lenses, corneal surgery, ablative procedures for trigeminal neuralgia, and systemic diseases including type 2 diabetes mellitus, multiple sclerosis, and leprosy. Clinically, its primary characteristic is the absence of pain [12]. As such, previous corneal surgery, previous trauma, abuse of topical medications, and prolonged use of contact lenses are among the antecedents requiring investigation. The topical medications that have been observed to be associated with the development of NK are anesthetics, antibiotics (sulfacetamide), antivirals (trifluridine), beta-blockers (timolol and betaxolol), steroids, and NSAIDs (diclofenac) by altering epithelial recovery [2, 3]. Although diverse symptoms have been reported for NK contingent on the triggering antecedents, the most common include dry eye symptoms in conjunction with reduced visual acuity [13]. A clinical history is critical for diagnosing NK, with physicians intentionally looking for antecedents associated with trigeminal lesions, ulcers, and decreased corneal sensitivity [14]. Nonetheless, a more comprehensive evaluation can include physical examination and the use of various tests, such as the sensitivity test performed by touching the central and peripheral portions of the cornea using the Cochet–Bonnet anesthesiometer to locate and quantify the loss of corneal sensitivity in response to stimulation with a nylon thread (< 5 mm indicates clinically significant hyposensitivity) [15]. The clinical classification of NK is based on changes in the corneal epithelium. There have been several classifications for this condition; nonetheless, the most recent one is that proposed by Dua et al. [16]. The severity of NK has been associated with corneal sensory loss, with its treatment depending on its staging and on improvements in the quality of the corneal epithelium based on the use of artificial tears and treatment of the underlying disease; however, there are currently multiple experimental treatments [17].

The current study aimed to identify the prevalence of NK in the Hispanic population. Moreover, we wanted to determine the main clinical characteristics of patients with NK upon receiving ophthalmological clinical care, the underlying diseases presented by patients with NK upon diagnosis, the staging of the disease upon diagnosis, and correlations among the obtained data to identify possible associations.

## Methods

**Fig. 1:**
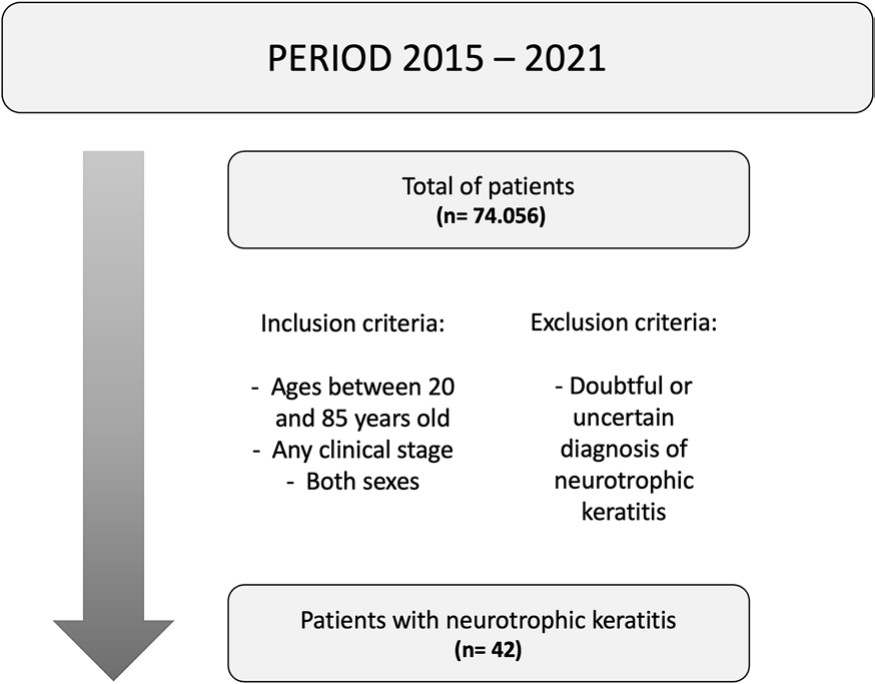
Criteria used for patient selection

An observational, descriptive, and retrospective cross-sectional study was conducted by collecting data from the medical records of patients diagnosed with NK at the Institute of Ophthalmology and Visual Sciences of Hospital Zambrano-Hellion and the Integral Health Center of the Santos y de la Garza Evia foundation (CAM) from March 2015 to November 2021. Male and female patients between 20 and 85 years who were diagnosed with NK of any clinical stage were included. Those with a doubtful or uncertain diagnosis of NK were excluded (Fig. 1). Information collected from the medical records of the patients diagnosed with NK included age, sex, medical history, symptoms, and clinical staging according to Mackie’s clinical staging system. For clinical purposes, those with stage 1, and 2 disease according to Mackie’s clinical staging system were grouped into the superficial corneal alterations group, whereas those with stage 3 diseases were grouped into the corneal ulcer/perforation group.

General descriptive analyses were performed using measures of central tendency, whereas comparative analyses were performed using Student's *t*-test or Mann–Whitney for quantitative data and Fisher's test or *χ*^2^ distribution for qualitative data. For the evaluation of normality, the Shapiro–Wilk test was performed. All analyses were conducted using IBM SPSS version 26, with a *p* value of < 0.05 indicating statistical significance.

## Results

From 2015 to 2021, a total of 74,056 patients were treated at the designated hospitals, among whom 42 were diagnosed with NK (Fig. 1). Accordingly, we found that NK had a prevalence of 5.67 (95% CI, 3.95–7.38) per 10,000 cases. The mean age of the included patients was 59 years, with NK occurring more frequently in males (59%) and those with corneal alterations (66.7%). The most frequent antecedents were use of topical medications (90%), presence of type 2 diabetes mellitus (40.5%), and systemic arterial hypertension (26.2%). Within the group of people with a history of using topical medications, chronic use of NSAIDs (68%), use of steroids (31%), and antivirals (13%) were observed. Clinical characteristics more frequently associated with NK were visual acuity deficiency (81%), followed by foreign body sensation (50%) (Table 1).

**Table 1:** Clinical characteristics of the study population

Clinical characteristics		
	M	SD
Age (years)	59	17.21
	*n* (42)	%
*Sex*		
Female	17	40.50
Male	25	59.50
*Clinical staging*		
I	19	45.20
II	9	21.40
III	14	33.30
*Severity*		
Corneal ulceration and/or perforation	14	33.30
Superficial corneal alterations	28	66.70
*Diseased eye*		
Right	15	35.70
Left	18	42.90
Both	9	21.40
Diabetes mellitus	17	40.50
Systemic arterial hypertension	11	26.20
Previous corneal surgery	8	19.00
Topic medicine	38	90.50
Contact lenses	3	7.10
Herpes virus infection	8	19.00
Eye redness	13	31.00
Without ocular pain	33	78.60
Visual acuity deficiency	34	81.00
Foreign body sensation	21	50.00
Constant tearing	8	19.00

*M,*—mean, *SD*—standard deviation

A higher proportion of male patients had corneal alterations, whereas a higher proportion of female patients had corneal ulcerations and/or perforation. Type 2 diabetes mellitus was observed more frequently in patients with corneal alterations than in those with corneal ulcerations and/or perforation. Among the clinical characteristics, the absence of ocular pain and visual acuity deficiency were observed most frequently (Table 2).

**Table 2:** Comparison between clinical characteristics and corneal alterations of the study population

Clinical characteristics	Corneal ulceration and/or perforation	Superficial corneal alterations	*p* value
	M, SD	M, SD	
Age (years)	51.93 ± 20.1	62.54 ± 14.71	0.0587
	*n* (%)	*n* (%)	
*Sex*			
Female	8, 57.14	9, 32.14	0.1836
Male	6, 42.86	19, 67.86	0.1836
*Clinical staging*			
I	0, 0	19, 67.86	
II	0, 0	9, 32.14	
III	14, 100	0, 0	
*Diseased eye*			
Right	6, 42.86	9, 32.14	0.5159
Left	7, 50	11, 39.29	0.5298
Both	1, 7.14	8, 28.57	0.2302
Diabetes mellitus	2, 14.29	15, 53.57	0.0204
Systemic arterial hypertension	1, 7.14	10, 35.71	0.0668
Previous corneal surgery	1, 7.14	7, 25	0.2328
Topic medicine	14, 100	24, 85.71	0.2829
Contact lenses	2, 14.29	1, 3.57	0.2537
Herpes virus infection	4, 28.57	4, 14.29	0.4064
Eye redness	6, 42.86	7, 25	0.298
Without ocular pain	10, 71.43	23, 82.14	0.4508
Visual acuity deficiency	11, 78.57	23, 82.14	0.9999
Foreign body sensation	6, 42.86	15, 53.57	0.7442
Constant tearing	4, 28.57	4, 14.29	0.4064

*M,*—mean, *SD*—standard deviation

## Discussion

NK is a disease with a prevalence of < 5 cases/10,000 population, 4.2 cases/10,000 inhabitants to be more precise. We note, however, that the exact incidence and prevalence rates have yet to be determined, with the aforementioned estimates seemingly overestimating the actual figures [3]. In fact, the mentioned prevalence rate was estimated and extrapolated from available data regarding conditions most associated with NK, being reported figures reaching as low as 1.6 cases/10,000 inhabitants with respect to estimates using herpetic keratitis (1.22/10,000 inhabitants) and postoperative damage to the trigeminal nerve (0.02/10,000 inhabitants) [2]. In our population the observed prevalence was 5.67 (95% CI, 3.95–7.38) per 10,000 cases. Current estimates show that on average, 6% of patients with herpetic keratitis develop NK, showing a prevalence of 149/10,000 inhabitants [4], and that 12.8% of those with herpes zoster keratitis develop the same, showing a prevalence of 26/100,000 inhabitants [5]. Unfortunately, no evidence have been available to date regarding the incidence and/or prevalence of other common associated conditions, such as chemical burns, type 2 diabetes mellitus, and prolonged contact lens use, as well as less frequently associated causes, such as intracranial masses, space occupants, multiple sclerosis, or leprosy [12, 18]. A recent retrospective, observational study at an ophthalmology center in Paris, France that screened more than 300,000 patients reported an incidence of 11/10,000 patients [19]. Our findings echo those reported in previously published studies. Likewise, our findings regarding age were also consisted with reported data. Importantly, we found that the most frequently occurring antecedent was the use of topical medications, highlighting the need for physicians to focus their attention toward this antecedent given that such patients could be at higher risk for developing NK.

Any condition that alters the innervation of the trigeminal nerve toward the cornea at any level of this circuit, from the nucleus to the free nerve endings, can eventually cause NK, an entity with specific diagnostic characteristics that are completely independent of the basic medical history of the patient at that time [3]. These etiologies can include conditions as diverse as those concerning ocular surface diseases, systemic pathologies, and both peripheral and central nerve damage [2, 20]. Both the herpes simplex and herpes zoster viruses have been identified as the most common causes of NK, with proportions ranging from 27 to 32% of cases [6–8]. In our population, we found a higher proportion of herpes virus infection, which accounted for 19% of the patients diagnosed with NK. It is important to mention the relevance of the finding of herpes virus infections, since being asymptomatic, most of them go unnoticed, causing recurrent lesions to corneal tissue, culminating in the development of NK. Other local conditions associated with this disease include chemical, thermal, and traumatic ocular burns, the use of contact lenses, corneal dystrophies, and the chronic use of topical medications such as anesthetics, beta-blockers, antivirals, antibiotics, non-steroidal anti-inflammatory drugs, and glaucoma treatment [2, 17]. We observed an important history of drug abuse, specifically NSAIDs, steroids, and antivirals, which can exacerbate the development of NK along with pre-existing pathologies. Surgical procedures that involve the cornea, such as refractive surgery, keratoplasty and its derivatives, and cataract surgery, have also been associated with corresponding alterations in the trigeminal innervation of the treated area [9, 10, 20]. In addition, therapeutic surgeries for trigeminal neuralgia, such as microvascular decompression, balloon compression, radiofrequency thermocoagulation, and gamma laser knife radiosurgery, have been strongly associated with NK.

NK is a chronic disease with an insidious evolution, which has been of interest due to the diverse clinical characteristics it presents. In our study population, we found that 40% of the patients with NK had a history of type 2 diabetes mellitus, which should be considered when treating this disease. Regarding the progression of the disease, the variety of clinical presentations needs to be accounted for. Our findings showed that the absence of eye pain, redness, and constant tearing were clinical symptoms associated with disease progression, which is consistent with that reported in the literature [20]. Thus, physicians need to proactively consider the possibly of NK when encountering the clinical symptoms described earlier. One limitation of the current study is our sample size, which, although greater than expected, could be increased further had the registry of patients from both public and private hospitals within the locality been analyzed.

## Conclusion

NK is an underdiagnosed disease with a broad clinical spectrum. The antecedents identified herein were consistent with those reported as risk factors in previous studies. Given the lack of reports regarding the prevalence of the disease within the studied geographical area, we expect prevalence rates to increase over time as physicians actively seek to diagnose the disease.

## Abbreviations

ACh: Acetylcholine
NK: Neurotrophic keratitis
SP: Substance P
